# Enhanced Biofilm Formation by *Escherichia coli* LPS Mutants Defective in Hep Biosynthesis

**DOI:** 10.1371/journal.pone.0051241

**Published:** 2012-12-28

**Authors:** Ryoma Nakao, Madeleine Ramstedt, Sun Nyunt Wai, Bernt Eric Uhlin

**Affiliations:** 1 Department of Molecular Biology and Laboratory for Molecular Infection Medicine Sweden (MIMS), Umeå Centre for Microbial Research (UCMR), Umeå University, Umeå, Sweden; 2 Department of Chemistry, Umeå University, Umeå, Sweden; 3 Department of Bacteriology, National Institute of Infectious Diseases, Tokyo, Japan; University of Würzburg, Germany

## Abstract

Lipopolysaccharide (LPS) is the major component of the surface of Gram-negative bacteria and its polysaccharide portion is situated at the outermost region. We investigated the relationship between the polysaccharide portion of LPS and biofilm formation using a series of *Escherichia coli* mutants defective in genes earlier shown to affect the LPS sugar compositions. Biofilm formation by a deep rough LPS mutant, the *hldE* strain, was strongly enhanced in comparison with the parental strain and other LPS mutants. The *hldE* strain also showed a phenotype of increased auto-aggregation and stronger cell surface hydrophobicity compared to the wild-type. Similar results were obtained with another deep rough LPS mutant, the *waaC* strain whose LPS showed same molecular mass as that of the *hldE* strain. Confocal laser scanning microscopy (CLSM) analysis and biofilm formation assay using DNase I revealed that biofilm formation by the *hldE* strain was dependent on extracellular DNA. Furthermore, a loss of flagella and an increase in amount of outer membrane vesicles in case of the *hldE* strain were also observed by transmission electron microscopy and atomic force microscopy, respectively. In addition, we demonstrated that a mutation in the *hldE* locus, which alters the LPS structure, caused changes in both expression and properties of several surface bacterial factors involved in biofilm formation and virulence. We suggest that the implication of these results should be considered in the context of biofilm formation on abiotic surfaces, which is frequently associated with nosocominal infections such as the catheter-associated infections.

## Introduction

Some surface structures of bacterial cells, such as flagella, curli fibers, type I fimbriae, and Ag43, are involved in biofilm formation of *Escherichia coli* [1], [2]. In addition, conjugative plasmids provide aggregative properties that stimulate biofilm production in *E. coli* [3]. Biofilms are extraordinarily resistant to conventional antibiotics, biocides and host immune defenses via the “bulky shields” reinforced by extracellular polymeric substances (EPS) [4]. One major component of the EPS in *E. coli* is colanic acid, an exopolysaccharide, which forms a protective capsule surrounding the bacterial cell and also sustains the biofilm architecture [5]. Extracellular DNA (eDNA) plays a significant role in biofilm formation, as revealed by recent studies in several bacteria including *E. coli* [6], [7], [8], [9], [10], [11], [12]. The eDNA in biofilms functions as a cell-to-cell interconnecting compound for *Pseudomonas aeruginosa* [6].

In most Gram-negative bacteria, lipopolysaccharide (LPS) is one of the major constituents of the outer leaflet of the outer membrane and it provides the structural integrity of the outer membrane. In addition, LPS in many species interacts with a biotic or an abiotic surface because of its location at the front line between the bacterium and its environment [13], [14], [15], [16], [17], [18], [19], [20], [21]. LPS consists of three distinct components: the lipid A, which is the hydrophobic portion of the molecule anchored in the outer membrane; the O-antigen extending from the cell to the external environment; and the core oligosaccharide (core OS), which links the O-antigen to the lipid A. The main virulence determinant of LPS resides in the lipid A, which is recognized by the cell membrane protein TLR4/MD2 receptor as part of the innate immune response [22], [23]. LPS triggers the release of many inflammatory cytokines, in particular TNFα and interleukin-1β, and it has been implicated as the etiological agent of a variety of pathologies such as septic shock, organ failure and death [24]. The O-antigen portion of LPS is highly variable and antigenic, and therefore LPS can elicit a strong immunogenic response. On the other hand, core OS *per se* is not generally considered a virulence factor. Importantly, the structural variation of core OS, especially the inner (lipid A-proximal) core, is limited in sharp contrast to that of O-antigen [25]. The LPS inner-core OS in *E. coli* and *Salmonella* is composed of two 3-deoxy-D-*manno*-oct-2-ulosonic acids (Kdo) and three L-*glycero*-D-*manno*-heptose (Hep), called HepI, HepII, and HepIII, in the order from the proximal site. The composition of the inner-core is conserved across genera. Therefore, the possibility of targeting the core OS for general therapeutic application against Gram-negative bacterial infection has been extensively investigated 26,27,28,29,30,31. Although inhibition of Kdo biosynthesis is usually lethal for bacteria, a defect in Hep biosynthesis results in a viable bacterial cell with a characteristic phenotype, referred to as the “deep rough” phenotype of *E. coli* and *Salmonella* [25]. The genes related to the core biosynthesis are encoded by the chromosomal *waa* (formerly *rfa*) locus that consists of three operons. One of the *waa* genes, *hldE* (formerly *waaE* or *rfaE*) encodes the HepI transferase, which also has been considered as a candidate for developing therapeutic agents against infectious diseases [30] because it encodes an enzyme involved in biosynthesis of Hep present in the LPS inner-core. HldE is a bifunctional protein with two distinct functional domains, an N-terminal region with homology to the ribokinase superfamily (HldE1 domain) and a C-terminal region with homology to the cytidylyltransferase superfamily (HldE2 domain) [32]. Some reports have shown that deep rough LPS core mutants of Enterobacteriaceae; *waaG*, *rfaH*, and *waaQ* mutants of *E. coli* and the *waaG* mutant of *Salmonella* Typhimurium; failed to colonize the intestine [33], [34], [35]. However, it has not been systematically investigated yet what role the LPS structure in these species might play in biofilm formation on an abiotic surface.

Outer membrane vesicles (OMVs) are released into the surrounding environment by Gram-negative bacteria during bacterial growth, and the maximum OMV production occurs during the end of the logarithmic growth phase. Therefore, OMVs are not products of cell lysis or cell death. The released OMVs have been found in planktonic culture and in biofilms [36]. OMVs range in size from 20 to 250 nm in diameter and contain not only components of the outer membrane, such as lipopolysaccharide (LPS), outer membrane proteins, and phospholipids, but also periplasmic proteins and peptidoglycan because OMVs entrap some of the underlying periplasmic proteins and fragments of the cell wall when they are extruded from the cell surface. In addition, it has also been shown that OMVs contain DNA [37], which seems to be derived from both cytoplasmic DNA from bacterium itself and eDNA from lysed cells. It is however not clear how the cytoplasmic DNA traffics into the OMVs and how the eDNA is associated with the OMVs.

In the present study, we aimed at investigating the relationship between the polysaccharide portion of LPS and biofilm formation on an abiotic surface. Analysis of a series of non-polar LPS mutants of *E. coli* revealed that deep rough LPS mutants increased biofilm formation. Further analysis demonstrated that the *hldE* mutation caused pleiotropic effects; *i.e*.; changes in bacterial auto-aggregation, flagellar expression, and OMV production as well as biofilm formation of the *E. coli* strain. Our results also suggested that the mechanism of biofilm formation was strongly associated with eDNA. The significance of this phenomenon is discussed from the view of biofilm formation against an abiotic surface, which is frequently associated with nosocomial infection caused by catheterizaion.

## Materials and Methods

### Bacterial strains, plasmids, and culture conditions

The bacterial strains and plasmids used in this study are listed in Table 1. *E. coli* strains were grown in LB broth or on LB agar plates. Carbenicillin, chloramphenicol, kanamycin, and spectinomycin were supplemented at 50, 25, 50 and 50 μg/ml, respectively, when required.

**Table 1:** *E. coli* strains and plasmids used in this study.

*E. coli* strain or plasmid	Relevant genotypes, phenotypes or selective marker^a^	Source and/or description
BW25113	wild type, K12 strain, *lacI* ^q^ *rrnB*_T14_ Δ*lacZ*_WJ16_ *hsdR514* Δ*araBAD*_AH33_ Δ*rhaBAD*_LD78_.	NIG collection (Japan)
RN101	Δ*waaC*, BW25113 derivative, Heptose-less LPS. *The kan* cassette was removed by FLP flippase carried by pCP20.	This study
RN102	Δ*hldE*, BW25113 derivative, Heptose-less LPS. The *kan* cassette was removed by FLP flippase carried by pCP20.	This study
RN103	Δ*waaF*, BW25113 derivative, inner-core mutant LPS contains 2 KDO and 1 heptose. The *kan* cassette was removed by FLP flippase carried by pCP20.	This study
RN104	Δ*waaG*, BW25113 derivative, core mutant LPS which lacks outer-core. The *kan* cassette was removed by FLP flippase carried by pCP20.	This study
RN105	Δ*waaL*, BW25113 derivative, No O-antigen LPS. The *kan* cassette was removed by FLP flippase carried by pCP20.	This study
RN106	Δ*waaP*, BW25113 derivative, dephosphorylated LPS. The *kan* cassette was removed by FLP flippase carried by pCP20.	This study
RN107	Δ*galE*, BW25113 derivative, LPS lacking galactose. The *kan* cassette was removed by FLP flippase carried by pCP20.	This study
RN108	Δ*agn43*, BW25113 derivative, autotransporter antigen 43 (Ag43). The *kan* cassette was removed by FLP flippase carried by pCP20.	This study
RN109	Δ*hldE*Δ*agn43::kan*, BW25113 derivative. Km^r^.	This study
RN110	*flhD::*Tn*5*, BW25113 derivative, regulator of the flagellar regulon. flagella deficient, Km^r^.	This study
PRG11	Δ*agn43*, MG1655 derivative, autotransporter Ag43 deficient.	Cabrer-Panes et al., unpublished data
PRG31	Δ*oxyR*, MG1655 derivative, derepression of Ag43 by a loss of the global transcriptional regulator, OxyR.	Cabrer-Panes et al., unpublished data
KP7600	wild type, K12 strain, *F^−^ lacI^q^ lacZ*Δ*M15 galK2 galT22* λ*^−^ in (rrnD-rrnE)1*, W3110 derivative	NIG collection (Japan)
NEB turbo	used for cloning, F' *proA* ^+^ *B* ^+^ *lacI* ^q^ Δ*lacZM15*/*fhuA2* Δ (*lac-proAB*) *glnV gal R*(*zgb-210*::Tn*10*)Tet^s^ *endA1 thi-1* Δ (*hsdS-mcrB*)*5*	New England Biolabs (Ipswich, MA)
pNTR-SD	ColE1 derivative, 8.3kb, Amp^r^	NIG collection (Japan)
pNT3(*hldE*)	pNTR-SD derivative containing the wild type *hldE* gene under *tac* promoter, utilized for complementation of the *hldE* gene mutant, Amp^r^	NIG collection (Japan)
pKD4	Template plasmid for the amplificationof the *kan* cassette, Km^r^, Cb^r^.	[55]
pKD46	Red recombinase expression vector. Temperature sensitive (*ts) plasmid which* replicates at 30°C, Cm^r^ Cb^r.^	[55]
pCP20	*ts* (replicates at 30°C) plasmid bearing the *flp* recombinase gene, Cm^r^ Cb^r.^	[53]
pMF19	low copy number expression vector, pEXT21, derivative containing the *wbbL* gene, which can restore the expression of O-antigen in rough LPS, 10.8kb, Spec^r.^	[56]
pMF19Δ*wbbL*	removed *wbbL* gene from pMF19, vector control of pMF19. 9.8kb, Spec^r.^	This study

a Cb^r^: carbenicillin resistant, Cm^r^: chloramphenicol resistant, Km^r^: kanamycin resistant, Spec^r^; spectinomycin resistant.

### LPS extraction and silver stain

LPS of BW25113, RN105, and RN107 was isolated by a LPS extraction kit (iNtRON Biotechnology, Kyunggi-do, Korea). LPS of RN101, RN102, RN103, RN104, and RN106 was isolated by phenol-chloroform-petroleum ether (PCP) methods as described by Galanos et al [38]. Purified LPS was resuspended with 1× loading buffer (50 mM of Tris-Cl (pH 6.8) containing 2% SDS, 13.5% glycerol, and 0.1 M dithiothreitol) for electrophoresis on a Tris-Tricine 15% sodium dodecyl sulfate (SDS)-polyacrylamide gel and was subjected to silver staining.

### Biofilm formation assay

Biofilm formation by *E. coli* was assayed as previously described [39] with some modifications. For biofilm analysis, 1×10^7^ CFU of *E. coli* in 100 μl of LB broth (1×10^8^ CFU/ml) was inoculated into the wells of a 96-well flat-bottom polystyrene microtiter plate (Corning 3595, New York, NY). Five-ml polystyrene tubes (Falcon 352058, BD labware, Franklin Lake, NJ) were also used to visualize biofilms. The bacterial strains were grown at 37°C for 48 hours under a static condition and the planktonic cells in liquid medium were discarded. The plate or tube was washed twice with distilled water and air-dried. Attached biofilms were stained with 0.1% crystal violet for 20 minutes. Then, the plates were rinsed twice with distilled water to remove excess stain and air-dried. In order to quantify the amount of biofilm on a 96-well plate, all stain associated with the attached biofilms was dissolved with 95% ethanol, then OD_595_ absorbance was measured using a microplate reader (Multiskan RC, ThermoFisher, Waltham, MA).

### Autoaggregation assay

Overnight-cultured bacteria were harvested by centrifugation (10,000× *g* for 2 minutes) and two ml of whole cells standardized at OD_600_ = 1 after suspension in PBS were placed in a 14-ml polyethylene tube (Falcon352057, BD labware) and incubated at 4°C under a static condition. The OD_600_ of the phase above the sediment by aggregation was recorded at different time points.

### Hydrophobicity assays

Hydrophobicity on the cell surface was determined by a hexadecane assay and by cryo-X-ray photoelectron spectroscopy (XPS) analysis. The hexadecane hydrophobicity assay is based on bacterial adhesion to hexadecane (specific gravity: 0.773), which has been described previously [40], and was performed with some modifications. In brief, bacteria were collected from an overnight culture and two ml of whole cells standardized at OD_600_ = 1 in PBS were placed into a 14-ml polyethylene tube (Falcon352057, BD labware). Two hundred μl of hexadecane (Sigma, St. Louise, MO) was added and the tube was vigorously vortexed for two minutes and subsequently incubated for 10 min at 15–24°C to allow for phase separation before the OD_600_ of the lower aqueous phase was measured. The percent hydrophobicity was calculated using the following formula: % hydrophobicity  =  [1− (OD_600_ after vortex/OD_600_ before vortex)] ×100. XPS analysis of bacterial samples were performed as described previously [41], as an alternative hydrophobicity assay. Bacterial samples were collected from fresh colonies on plates grown at 37°C for 17 hrs. The bacteria were washed twice with PBS and then bacterial pellets were placed on a sample holder with a grid for XPS analysis. The sample holder was placed on the pre-cooled transfer rod (−160°C) in the loading chamber and the pellet was allowed to quickly freeze before the pressure was decreased. XPS spectra were collected from the frozen pellet (kept at −165°C throughout the measurement) using a Kratos Axis Ultra DLD photoelectron spectrometer using monochromated Al Kα source operated at 150 W. An analyzer pass energy of 160 eV was used for acquiring wide spectra and 20 eV for acquiring individual photoelectron lines. The spectrometer charge neutralizing system was used to compensate for sample charging during the measurement, and the binding energy scale was referenced to the C1s aliphatic carbon peak at 285.0 eV. By analyzing the bacteria in the form of quick-frozen wet pellets, alterations in the bacterial cell wall due to dehydration and drying is minimized [42].

### SDS-PAGE and Western blot analysis

From one ml of overnight-cultured bacteria, whole cells and supernatants were harvested by centrifugation (10,000× *g* for 2 minutes). The supernatant samples were concentrated by precipitation with 10% trichloroacetic acid and two washes with 80% acetone chilled at −20°C. After the cell number was standardized, whole cells or supernatants were by resuspended with 1× loading buffer (50 mM of Tris-Cl (pH 6.8) containing 2% (w/v) SDS, 0.1% bromophenol blue, 10% glycerol, and 1.7 M 2-mercaptoethanol) for SDS-polyacrylamide gel electrophoresis (SDS-PAGE). Western blot analysis was carried out by standard methods. Rabbit antisera against *E. coli* FliC [43], [44], DsbA [45], Crp (this laboratory), Ag43 [46], OmpC (this laboratory), and OmpA (this laboratory) were used at 1∶2,000, 1∶5,000, 1∶3,000, 1∶5,000, 1∶2,000, and 1∶50,000 dilutions for Western blot, respectively. The O16 specific antiserum was purchased from Statens Serum Institut Diagnostika (Hillerød, Denmark) and used at 1∶200 dilution for Western blot. Horseradish peroxidase (HRP)-labeled anti-rabbit Ig antibody (GE Healthcare Bio-Sciences) diluted at 1: 20,000 was used as the secondary antibody for all Western blot experiments done in this study. Chemiluminescence was developed by ECLplus (GE Healthcare Bio-Sciences) and visualized by Chemidoc (Bio-rad) and Quantity One system (Bio-rad) or by exposure on X-ray film. The band densities of the signals in Western blot analysis were quantified using densitometric scanning (CS analyzer 1.0; ATTO Co., Tokyo, Japan).

### Transmission electron microscopy (TEM)

For transmission electron microscopy (TEM) examinations, *E. coli* biofilms were developed on 96-well polystyrene plates by the same procedure as for the biofilm formation assay. After two washes with distilled water, attached cells were collected by thoroughly pipetting with 10 mM Tris-Cl (pH 7.4) with 10 mM MgCl_2_. Cells were allowed to adhere to formvar-coated grids for three minutes at 15–24°C, then negatively stained with 1% sodium silicotungstate. TEM was performed using a JEOL 1230 (JEOL Ltd., Tokyo, Japan).

### Confocal laser scanning microscopy (CLSM)

To prepare the biofilm sample for CLSM analysis, biofilms were grown on cover glass (φ12 mm #1; Menzel GmbH&Co., Braunschweig, Germany) placed in 6-well polystyrene cell culture plates (Nunc A/S, Roskilde, Denmark). Two ×10^8^ CFU of *E. coli* was added in two ml of LB broth per well and incubated at 37°C. After 48 hours, the cover glass was rinsed twice with PBS to remove any planktonic cells. Attached cells on the cover glass were stained with 1% acridine orange for 5 minutes or with a mixture of 5 μM SYTO 9 (Invitrogen, Carlsbad, CA) and 1 μM BOBO-3 (Invitrogen) for 20 minutes at 15–24°C, followed by two washes in PBS and one wash in distilled water. To visualize extracellular DNA (eDNA), the biofilms were also stained with SYTO 9 (Invitrogen) and BOBO-3 (Invitrogen), as described previously [47]. SYTO 9 allows visualization of cells, whereas BOBO-3 is a membrane-impermeable dye that binds to DNA and therefore specifically stains extracellular DNA [47], [48]. After the preparation, the samples were examined under a confocal laser scanning microscope, (Nikon confocal D-eclipse C1; (Nikon Instruments Inc., Melville, NY) or ZEISS LSM 710 (Carl-Zeiss), and images were processed by EZ-C1 software (Nikon Instruments Inc.) or LSM software ZEN (Carl-Zeiss, Oberkochen, Germany), respectively. SYTO 9 was excited at 488 nm argon laser line and fluorescence emission was detected between 500–530 nm. Acridine orange and BOBO-3 were excited at 561 nm He-Ne laser line and fluorescence emission was detected between 570–620 nm. Fluorescene signal of double labeled specimens and transmission images were acquired simultaneously.

### OMV preparation and atomic force microscopy (AFM)

OMVs were isolated from culture supernatants as previously described [49], with some modifications. After centrifugation at 2,975× *g* for 15 minutes at 4°C, the supernatant was filtered through a 0.45 μm Durapore PVDF filter (Millipore, Billerica, MA). OMVs were then collected by centrifugation at 150,000× *g* for 3 hours at 4°C in a 45 Ti roter (Beckman Instruments, Fullerton , CA). The pellets were resuspended with 20 mM Tris-Cl (pH 8.0) and used as the OMV preparation. The OMV preparations were diluted with ultrapure water and placed on a freshly cleaved mica surface. The samples were incubated at 15–24°C for 5 minutes, gently washed with ultrapure water and dried in a desiccator for at least 3 hours. Imaging was performed with a Nanoscope IIIa Atomic Force Microscope (Veeco Instruments) using tapping mode. The protein profile or amount of each OMV preparation was also examined by silver staining after PAGE or Bradford assay [50], respectively.

### Detection and quantification of eDNA

To obtain eDNA in the supernatant, 2-day-old culture supernatant was collected by centrifugation at 17,400× *g* for 5 minutes at 4°C. The supernatant was serially diluted with Tris (10 mM)- EDTA (5 mM) (pH 8.0) (TE) buffer. The amount of total dsDNA in the supernatant was measured by Quant-iT PicoGreen dsDNA Reagent and Kits (Invitrogen). *E. coli*-specific DNA was measured by PCR using oligonucleotides, atoS-f1 and atoS-r1. The PCR reaction mix contained 1 μl of diluted supernatant (template DNA), 5 μl of Premix Taq Ex Taq Version (Takara Bio Inc, Shiga, Japan), and 0.4 μM of each primer in a total volume of 10 μl. eDNA associated with the biofilms and intracellular DNA (iDNA) in the biofilm cells was also obtained and quantified as described previously [47], [51], with some modifications. Biofilms in the bottom of the culture tube with a statically grown 2-day-old 8 ml culture were collected by careful removal of the broth. Two hundred μl of TE buffer (10 mM Tris, 1mM EDTA [pH 8]) was added to the tube, and the biofilms were scraped from the surface using gentle pipetting. The scraped biofilms were transferred to a 1.5-ml tube and centrifuged at 17,400× *g* for 2 min at 4°C. The supernatant was removed and the pellets were vigorously resuspended with 200 μl of TE buffer, and the tubes were recentrifuged at the same conditions. The cell pellet and supernatant were used for isolation of iDNA and eDNA using DNeasy (Qiagen, Hilden, Germany), respectively. After isolation of iDNA and eDNA, both were suspended in 100 μl of TE buffer, and DNA concentrations were measured using a NanoDrop 1000 (Thermo Fisher Scientific, Waltham, MA). The results are given as the ratio of eDNA and iDNA in the biofilms. Samples of eDNA were also separated in a 1% agarose gel and visualized by staining with ethidium bromide.

### DNA manipulations

All DNA manipulations were carried out using standard methods [52]. The oligonucleotides used in this study are listed in Table S1. Insertion-deletion mutation was confirmed by PCR using specific primer pairs for each gene. The plasmid pCP20 was used for elimination of the *kan* cassette in the chromosome of deletion mutants from the NIG collection [53]. Insertion of an IS*5* element into the *wbbL* gene in the chromosomes of BW25113 and KP7600 was confirmed by PCR using oligonucleotides, wbbL-f and wbbL-r. pMF19Δ*wbbL* was constructed by the digestion of pMF19 with *Eco*RI followed by the self-ligation. To make a flagellar mutant of the strain BW25113, a Tn*5* insertion in the *flhD* gene was introduced by generalized transduction using P1 phage and the transductants were isolated by selection for kanamycin resistance [54]. An otherwise isogenic Δ*hldE*Δ*agn43* double mutant, RN109, was constructed from strain RN102 (Δ*hldE*) using the method described by Datsenko and Wanner [55]. Briefly, oligonucleotides agn43del-f and agn43del-r were used to amplify a *kan* cassette from a template plasmid, pKD4. The resulting PCR product was used to knock out the desired genes using the Red recmbinase system provided on the curable helper plasmid pKD46.

### Statistical analysis

Statistical analysis was performed using one-way analysis of variance (ANOVA) followed by the Dunnett's multiple comparison test or Mann-Whitney's U-test. *P*-values of 0.05 or less were considered to indicate statistical significance.

## Results

### The sugar composition of LPS has an effect on biofilm formation

**Figure 1:**
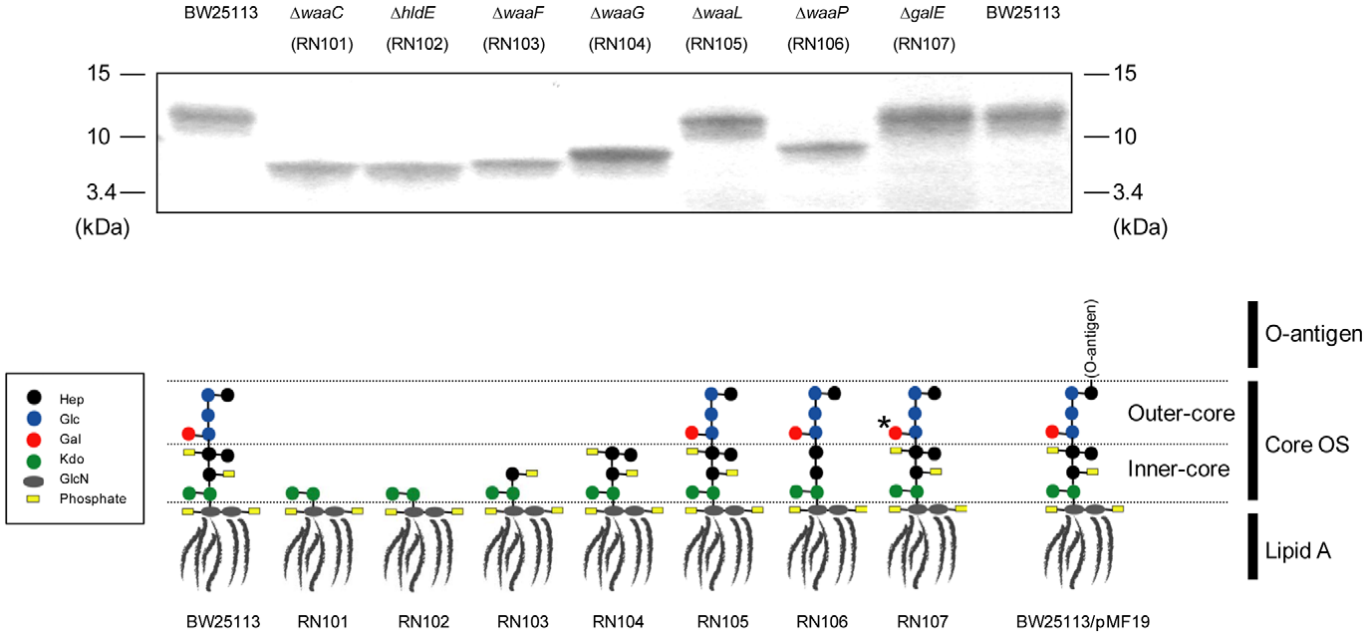
Generalized LPS structures in a series of *E. coli* LPS mutants used in this study. A silver-stained polyacrylamide gel after SDS-PAGE is shown in the upper panel. In the lower panel, the drawing of LPS structures especially highlights the core OS portion of LPS. Structures of the major glycoforms of core OS are based on a structural analysis of a K-12 strain, W3100 [59] and a *waaP* mutant of strain R1 [60]. The LPS core OS composition of the *hldE* strain (RN102) is the same as that of the *waaC* strain (RN101), probably because the lack of HldE protein results in arrest of ADP-Hep biosynthesis. It has been reported that in the *E. coli* R1 strain, inactivation of the *waaP* gene results not only in the loss of all phosphate groups on HepI and HepII, but also loss of HepIII [60]. We could not detect any difference in size of LPS between RN107 and BW25113 or RN105, although the loss of Gal (shown by an asterisk) was expected theoretically. Therefore, the LPS structure of RN107 was described based on the information of the SDS-PAGE gel in this study. A more precise analysis is required to fully understand the LPS structure of RN107. Each sugar or amino sugar of core OS is shown by a black (Hep), blue (Glc), red (Gal), green (Kdo), or gray (GlcN) circle. Phosphate groups (Phosphate or pyrophosphorylethanolamine) modified on sugar are shown by yellow boxes (P/PPEtN). Hep: L-*glycero*-D-*manno*-heptose, Kdo: 3-deoxy-D-*manno*-oct-2-ulosonic acid, GlcN: *N*-acetylglucosamine.

**Figure 2:**
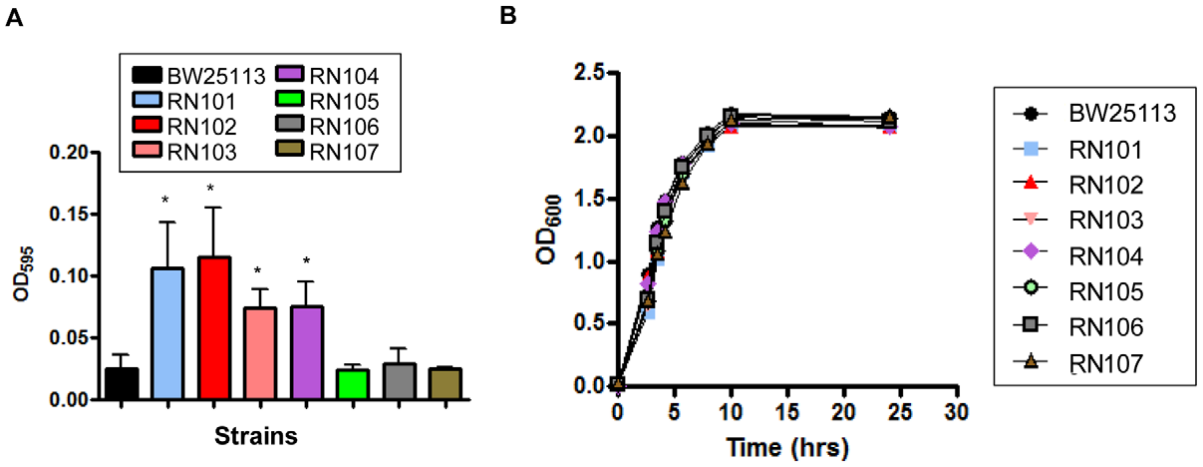
Biofilm formation and growth of a series of LPS mutants. (A) Biofilm formation by a series of core OS LPS mutants when compared to the parental strain, BW25113. The mean ± SD of results from 3 independent experiments are shown. Statistical analysis was performed using ANOVA. *P<0.05, against biofilm formation level of strain BW25113 (B) Growth of BW25113 and the LPS mutants. Each strain was grown in LB broth under shaking conditions at 37°C. Absorbance at OD_600_ was measured at different time points.

The aim of this study was to examine the biofilm formation of isogenic *E. coli* strains carrying LPS with different sugar compositions in the polysaccharide portion. To examine whether the sugar composition of core OS is involved in the biofilm formation of *E. coli*, a series of LPS mutants were compared with their parental strain, BW25113, which is a K-12 strain. In this study, the *waaC*, *hldE*, *waaF*, *waaG*, *waaL*, *waaP*, and *galE* genes were selected for mutation because this set of genes is involved in the assembly or modification of core OS or O-antigen ligation (Table 2). The LPS profiles of the wild type and the mutants on Tris-Tricine SDS-PAGE gel were visualized by silver staining and were described as generalized LPS structures (Fig. 1). As we expected, the *waaC* and *hldE* strains (RN101 and RN102) have same and smallest molecular mass (MM) of LPS among strains used in this study. The *waaF* strain (RN103) LPS showed slightly larger MM than that of the RN101 or RN102. The *waaG* strain (RN104) LPS showed further larger MM than the RN103. The *waaP* strain (RN106) LPS showed smaller or larger MM than that of BW25113 or RN104, respectively. However, the difference in MM between wild type and the *waaL* or *galE* strain (RN105 or RN107) was not observed on SDS-PAGE gel. We firstly examined the biofilm formation of each strain in 96-well microtiter plates. We observed that the biofilm formation was significantly increased in RN101, RN102, RN103, and RN104, in comparison with the parental strain (Fig. 2A), while all the strains in this experiment showed similar growth curves (Fig. 2B). In particular, two deep rough LPS mutants, the RN101 and RN102, showed the strongest biofilm formation among these strains (Fig. 2A). An *E. coli* K-12 strain often lacks the long O-antigen because of an IS*5* insertion interrupting the function of the *wbbL* gene, which encodes a rhamnosyltransferase involved in an addition of rhamnose to the O-repeat. We examined the effect of O-antigen on biofilm formation using two different K-12 strains, BW25113 and KP7600. PCR analysis of a region flanking the *wbbL* gene showed the presence of IS*5* in the *wbbL* gene in both BW25113 and KP7600 (data not shown). Restoration of O-antigen by introduction of the plasmid pMF19 containing the *wbbL* gene [56] into the two *E. coli* K-12 strains was confirmed by Western blot using O16 antiserum (Fig. S1). Both of the two O-antigen complemented strains were decreased in biofilm formation when compared to the vector control (Fig. S2). These data suggested that a difference in sugar composition of LPS affected the intensity of biofilm formation. In particular, we found that composition of inner-core OS is strongly associated with biofilm formation in *E. coli*.

**Table 2:** Function and phenotype expressed by mutation of the genes selected in this study.

Gene name	Alternative gene name(s)	Function in core OS assembly	Charater of LPS core
*waaC*	*rfaC*	LPS heptosyltransferease for HepI	Hep-deficient
*hldE*	*rfaE, waaE*	Heptose 7-P kinase/heptose 1-P adenyltransferase	Hep-deficient
*waaF*	*rfaF*	LPS heptosyltransfererase for HepII	KDO with one heptose
*waaG*	*rfaG*	LPS α1,3-glucosyltransferase for HexI	Intact 3 heptose but outer core-less
*waaL*	*rfaL*	LPS O-antigen ligase	Intact core
*waaP*	*rfaP*	LPS kinase for HepI phosphorylation	Core lacking HepIII as well as phosphoryl modifications on HepI and HepII
*galE*	-	UDP-galactose 4-epimerase	Intact core or core lacking galactose*

* LPS character of the RN107 was described based on the information of the SDS-PAGE gel in this study. A more precise analysis is required to fully understand the LPS structure of RN107.

### The RN102 strain shows strong auto-aggregation with an increase in hydrophobicity, loss of flagella, and enhancement of Ag43 expression

**Figure 3:**
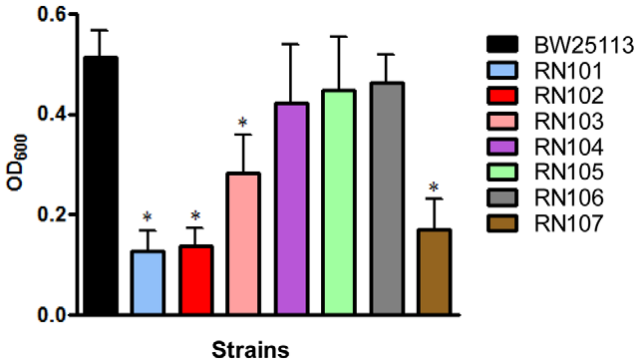
Autoaggregation phenotype by LPS mutants. Each strain standardized at OD_600_ = 1.0 in PBS was used for autoaggregation assay. The value at OD_600_ after an18-hour incubation is shown as the mean ± SD of results from three independent experiments. Statistical analysis was performed using ANOVA. *P<0.05, against autoaggregation level of strain BW25113.

**Figure 4:**
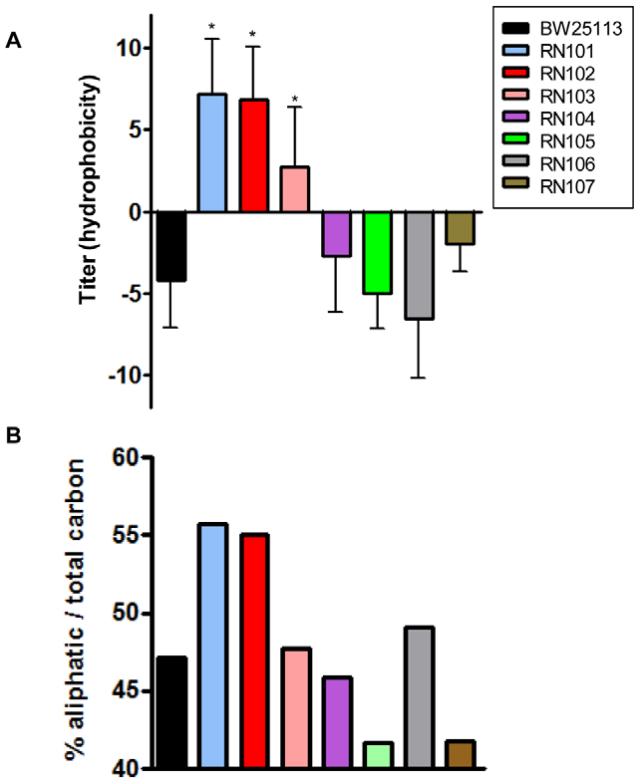
Surface hydrophobicity of LPS mutants. (A) Hydrophobicity assay using hexadecane. Each strain standardized at OD_600_ = 1.0 in PBS was used for a hexadecane hydrophobicity assay. Absorbance at OD_600_ in the water phase was quantified before and after a 10-min incubation. The percent hydrophobicity was calculated by the formula: % hydrophobicity  =  [1− (OD_600_ after vortex/OD_600_ before vortex)] ×100. The mean ± SD of results from 4 independent experiments are shown. Statistical analysis was performed using ANOVA. *P<0.05, against hydrophobicity of strain BW25113. (B) XPS data showing the percent of total carbon at the surface that is present in the form of aliphatic (non polar) carbon.

We examined auto-aggregation of the mutant strains. The RN101, RN102, RN103, and RN107 showed stronger auto-aggregation than the parental strain. In particular, the strains RN101 and RN102 were the two most auto-aggregative strains among the series of LPS mutants (Fig. 3). To further analyze this, we examined the hydrophobicity of the bacterial surface by a hexadecane assay. The strains RN101 and RN102 had increased hydrophobicity of their bacterial surfaces in comparison with the parental strain (Fig. 4A). Furthermore, in order to analyze the chemical property of the bacterial surface, we performed XPS analysis using frozen pellet samples from a series of LPS mutants. The ratio of aliphatic carbon to total carbon on the bacterial surface can be used as a measure of the presence of non-polar molecules or side groups. This ratio was compared among a series of LPS mutants. As expected, the level of auto-aggregation was influenced by a difference in chemical property of the bacterial surface. The ratios of the aliphatic carbon to total carbon in the RN101 and RN102 strains were higher than that of the parental strain (Fig. 4B). We also found that the aliphatic carbon peak level of the RN101 bacterial surface was restored to the wild type level by introduction of the *hldE* gene expression plasmid (pNT3(*hldE*)) (Fig. S3B). Results from both the hexadecane assay and XPS analysis suggested that composition of LPS core OS was important to retain the hydrophilicity on the bacterial surface.

**Figure 5:**
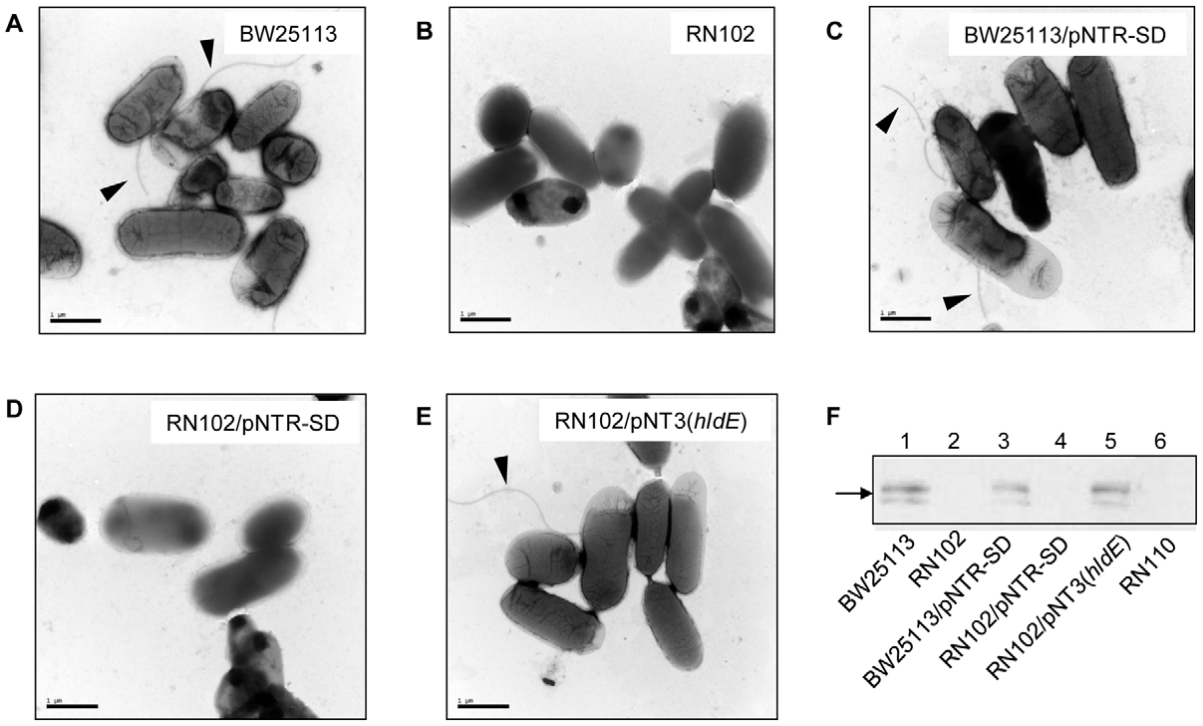
Loss of flagella in RN102. Fourty eight-hour-cultured biofilms were collected and analyzed by TEM, and by Western blot for FliC. (A–E) TEM images of the bacterial cells and the cell appendages are shown for strains (A) BW25113, (B) RN102, (C) BW25113/pNTR-SD, (D) RN102/pNTR-SD, and (E) RN102/pNT3(*hldE*). Flagella found in figures (A), (C), and (E) are shown by arrowheads. Representative electron-microphotographs of each strain are shown. A 1-μm-long bar is shown in the lower left corner. (F) Supernatants were collected from 48-hour bacterial cultures. Result of Western blot using anti-FliC antiserum is shown. Lanes; 1, BW25113; 2, RN102; 3, BW25113/pNTR-SD; 4, RN102/pNTR-SD; 5, RN102/pNT3(*hldE*); 6, RN110.

We also compared the cell morphology of RN102 with that of the wild type BW25113 by TEM (Fig. 5). Although flagella were found in BW25113 and BW25113/pNTR-SD strains (shown by arrowheads in Fig. 5A and C), flagella appeared absent in case of the RN102 and RN102/pNTR-SD strains (Fig. 5B and D). We confirmed the presence or absence of the flagella subunit protein FliC in the supernatants of BW25113 and BW25113/pNTR-SD strains or the RN102, RN102/pNTR-SD, and *flhD* mutant (flagella negative strain, RN110), respectively (Fig. 5F). Flagellar expression of the strain RN102 was restored by in-*trans* complementation of the *hldE* mutation (Fig. 5E and F, lane 5). We therefore examined the presence of the FliC in whole cells and supernatants of the series of LPS mutants (Fig. S4). Notably, in addition to RN102, the strains RN101 and RN103 also failed to express any detectable flagella protein in whole cells as well as supernatants. RN104 also drastically decreased the expression of the FliC. In contrast, the RN105 expressed a higher level of flagella protein. Besides, the expression levels of a major outer membrane protein, OmpC of the RN101, RN102, RN103, RN104, and RN106 decreased in comparison with the BW25113 strain and the expression level of the OmpC correlated with the amount of molecules of inner core OS, while the expression level of a cytoplasmic protein as a loading control, Crp, was comparable among all strain. The results suggest that the Hep of the inner-core is associated with the expressions of flagella and OmpC.

As it has been reported that Ag43 is involved in autoaggregation of *E. coli*, we also examined the expression level of Ag43 by Western blot analysis of cell extracts from BW25113, RN102, and their derivatives. The level of Ag43 protein in RN102 was clearly higher than in the wild-type strain and almost reached the level of an Ag43 de-repressed strain carrying an *oxyR* mutation (Fig. S5A). The RN102 restored the normal expression of Ag43 by the introduction of the pNT3(*hldE*) (Fig. S5A). To examine whether the higher Ag 43 expression level of the RN102 is associated with the autoaggregation and biofilm formation, a *hldE Agn43* double mutant, RN109, was constructed and used for the autoaggregation and biofilm formation assays (Fig. S5B, C, and D). RN109 showed similar properties of autoaggregation as well as biofilm formation to RN102. These results suggest that alteration of inner core OS composition also affects the Ag43 expression, however, the increased Ag43 expression is independent of enhancement of autoaggregation and biofilm formation in the RN102.

### Biofilm formation by the strain RN102 is enhanced by extracellular DNA

**Figure 6:**
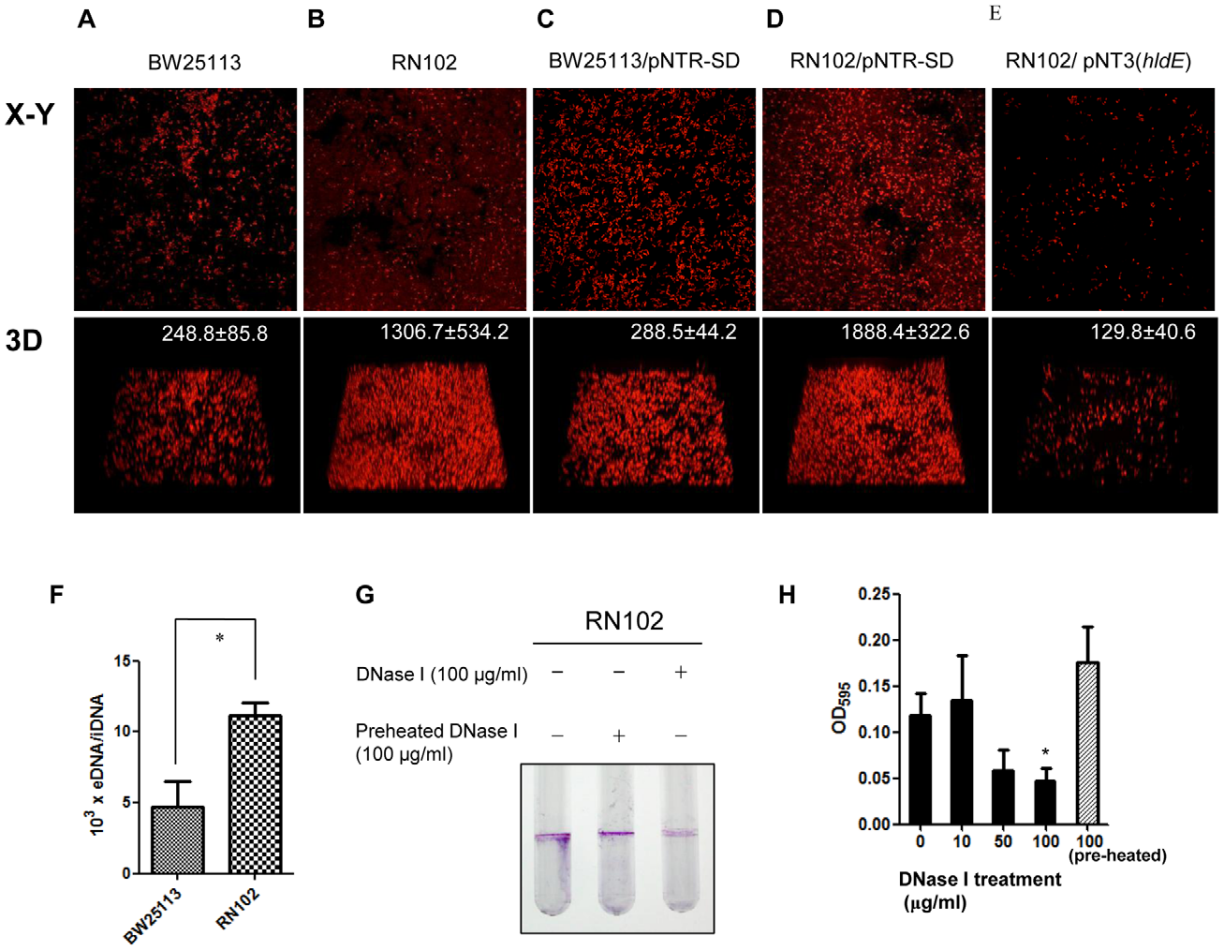
Contribution of eDNA to biofilm structure formed by RN102. (A–E) CLSM images of biofilms formed by strains: (A) BW25113 (B) RN102, (C) BW25113/pNTR-SD, (D) RN102/pNTR-SD, and (D) RN102/pNT3(*hldE*). Images of biofilms stained with acrydine orange are shown as digital CLSM images. In each strain, a section which has the largest sum of signals in the defined area (127.3 μm by 127.3 μm) among all X–Y sections is shown in the upper row (X–Y). The overview of biofilms in the same area of each X–Y section is shown as 3D image in the lower row (3D). The volume of each 3D image (μm^3^) in the area of the X–Y planes was quantified and the mean ± SD obtained from 3 different areas chosen at random are denoted in the upper-right corners. The data shown are representative microphotographs of two independent experiments. (F) Quantification of eDNA from BW25113 and RN102 strains. The bars represent the ratio of extracellular DNA to intracellular DNA (eDNA/iDNA). Results are shown as the mean ± SD from 3 independent experiments. *P<0.05. Stastical analysis was performed using Mann-Whitney's U-test. (G and H) Effect of DNase I on biofilm formation by the RN102 in a clear test tube (G) and as quantified in a 96-well plate (H). The RN102 was grown in presence of different concentrations of DNase I or in presence of pre-heated DNase I or without DNase I for 48 hours under static conditions at 37°C. The mean ± SD of results from 3 independent experiments are shown. Statistical analysis was performed using ANOVA. *P<0.05, against the biofilm formation by RN102 without DNase I treatment.

The biofilm architectures of BW25113 and RN102 were further examined using confocal laser scanning microscopy (CLSM) and acridine orange staining, which is a cell-permeable fluorescent dye and specifically binds to nucleic acid. Biofilms were grown on cover glass placed at the bottom of polystyrene cell culture plates. Although BW25113 did not appear to form biofilms (Fig. 6A), the RN102 formed readily detected biofilms, which were relatively flat and had a volume of more than 1 mm^3^ for an area of 161.29 μm^2^ (Fig. 6B). Notably, acridine orange stained EPS with dark red as well as bacterial cells with bright red, suggesting that the EPS contained extracellular nucleic acid. We confirmed that high level of extracellular DNA (eDNA) in the biofilms of RN102 was observed by staining with a cell-impermeant nucleic acid binding dye, BOBO-3, which allows visualization of eDNA, whereas eDNA was hardly detected in the wild type (Fig. S6A). The amount of eDNA in the biofilms of RN102 was clearly greater than that of the wild type (Fig. 6F). We also quantified the eDNA and intracellular DNA (iDNA) amounts in the biofilms formed in the bottom of tubes of both the wild type and the RN102 strains. The ratio of eDNA to iDNA for the wild type and the RN102 strains are shown in Fig. 6F. The ratio of the RN102 strain was significantly higher than that of the wild type strain. The eDNA was also analyzed on 1% agarose gel (Fig. S6B). High molecular weight molecules migrating above 10 kb was detected in eDNA samples from the RN102 strain, but not from the wild type strain. A biofilm formation assay using clear test tubes showed that the RN102 also formed biofilms at the interface between the liquid and air phases (Fig. 6G). The introduction of pNTR-SD (vector control) into either BW25113 or the RN102 had no effect on the appearance of the biofilms formed in the bottom of the cultures (Fig. 6C and 6D). The *hldE*-complementation was successful, as the RN102/pNT3(*hldE*) strain did not form strong biofilms (Fig. 6E). In order to test whether eDNA contributed to the biofilm formation by the RN102, we examined the effect of DNase I on the biofilm formation. When DNase I was added to the 48-hour-cultured biofilms, there was no effect on the biofilm formation (data not shown). An addition of DNase I at a concentration of 100 μg/ml did not inhibit the cell viability (data not shown). However, addition of DNase I at a concentration of 100 μg/ml at the onset of the culturing effectively inhibited biofilm formation of strain RN102 both as assessed with the clear test tube and with the 96-well plate (Fig. 6G and 6H). The inhibitory effect of this DNase I treatment was estimated to 59.7% (Fig. 6H). These data demonstrated that eDNA was involved in biofilm formation by RN102.

### Pleiotropic effect of the *hldE* mutation on bacterial factors associated with outer membrane

**Figure 7:**
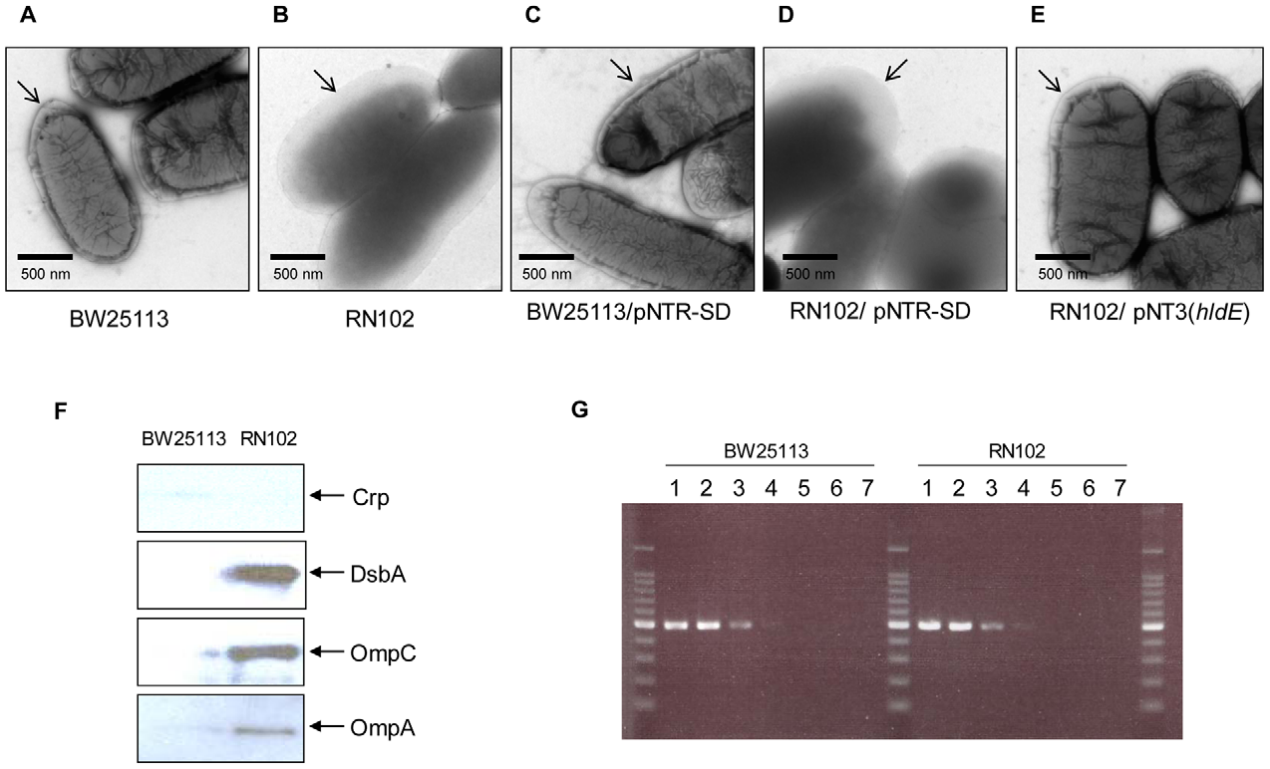
Loss of outer membrane integrity in strain RN102. Bacterial samples were collected from 48-hour-cultured biofilms for TEM analysis. TEM images of the bacterial cells and the cell appendages are shown for strains: (A) BW25113, (B) RN102, (C) BW25113/pNTR-SD, (D) RN102/pNTR-SD, and (E) RN102/pNT3(*hldE*). The outer membranes are indicated by arrows. Representative electron-microphotographs of each strain are shown. A 500-nm-long bar is shown in the lower left corner of each eclectron-micrograph. (F) Western blot analysis of supernatants from BW25113 and RN102. Supernatants were harvested by centrifugation from bacterial liquid culture grown for 48 hours under static conditions. Results of Western blot using anti-Crp, anti-DsbA, anti-OmpC, and anti-OmpA antisera are shown. (G) Supernatants from bacterial liquid cultures of BW25113 or RN102 grown for 48 hours under static conditions were serially diluted with TE. The diluted samples were used as template DNA for PCR using *E. coli atoS* gene-specific primer pairs. Lanes: 1, without dilution; 2, 10^−1^ dilution; 3, 10^−2^ dilution; 4, 10^−3^ dilution; 5, 10^−4^ dilution; 6, 10^−5^ dilution; 7, 10^−6^ dilution.

The TEM results also indicated a difference in cell surface morphology among these strains. The BW25113 strain retained a clear contour and contrast of the outer membrane when sodium silicotungstate was used as a negative staining dye (Fig. 7A). However, in case of RN102, the contour of the outer membrane was unclear and the cell was diffusely stained with sodium silicotungstate (Fig. 7B). Introduction of pNTR-SD into BW25113 or the RN102 did not affect the cellular appearance of either strain (Fig. 7C and 7D). The RN102/pNT3(*hldE*) retained a clear contour of the outer membrane (Fig. 7E), that is, in-*trans* complementation of the *hldE* mutation successfully restored the wild-type phenotype. These data suggested that the surface of RN102 might have altered membrane integrity and perhaps would be leaky. In order to investigate the effects of the *hldE* mutation on the membrane integrity of these strains, we investigated whether cytoplasmic or periplasmic proteins were present in the supernatant by Western blot analysis using antisera against Crp and DsbA, markers of cytoplasmic and periplasmic proteins, respectively (Fig. 7F). The results showed that strain RN102 released much more DsbA into the supernatant when compared to the parental strain, while neither of the strains released any Crp. This indicated that the integrity of the inner membrane of RN102 was similar to that of the parental strain, at least during the 48 hours of incubation under the conditions used. However, strain RN102 seemed to have lost some of the outer membrane integrity, which was consistent with our data from TEM analysis shown in Figure 7B. Furthermore, we compared the amount of DNA in the supernatant between the wild type and RN102. There was little difference in the amount of not only total dsDNA (data not shown), but also *E. coli*-specific DNA (Fig. 7G) between the two strains, arguing against an active release of cytoplasmic DNA to the extracellular milieu by cell lysis of RN102 and again indicating the retained integrity of the inner membrane of RN102.

**Figure 8:**
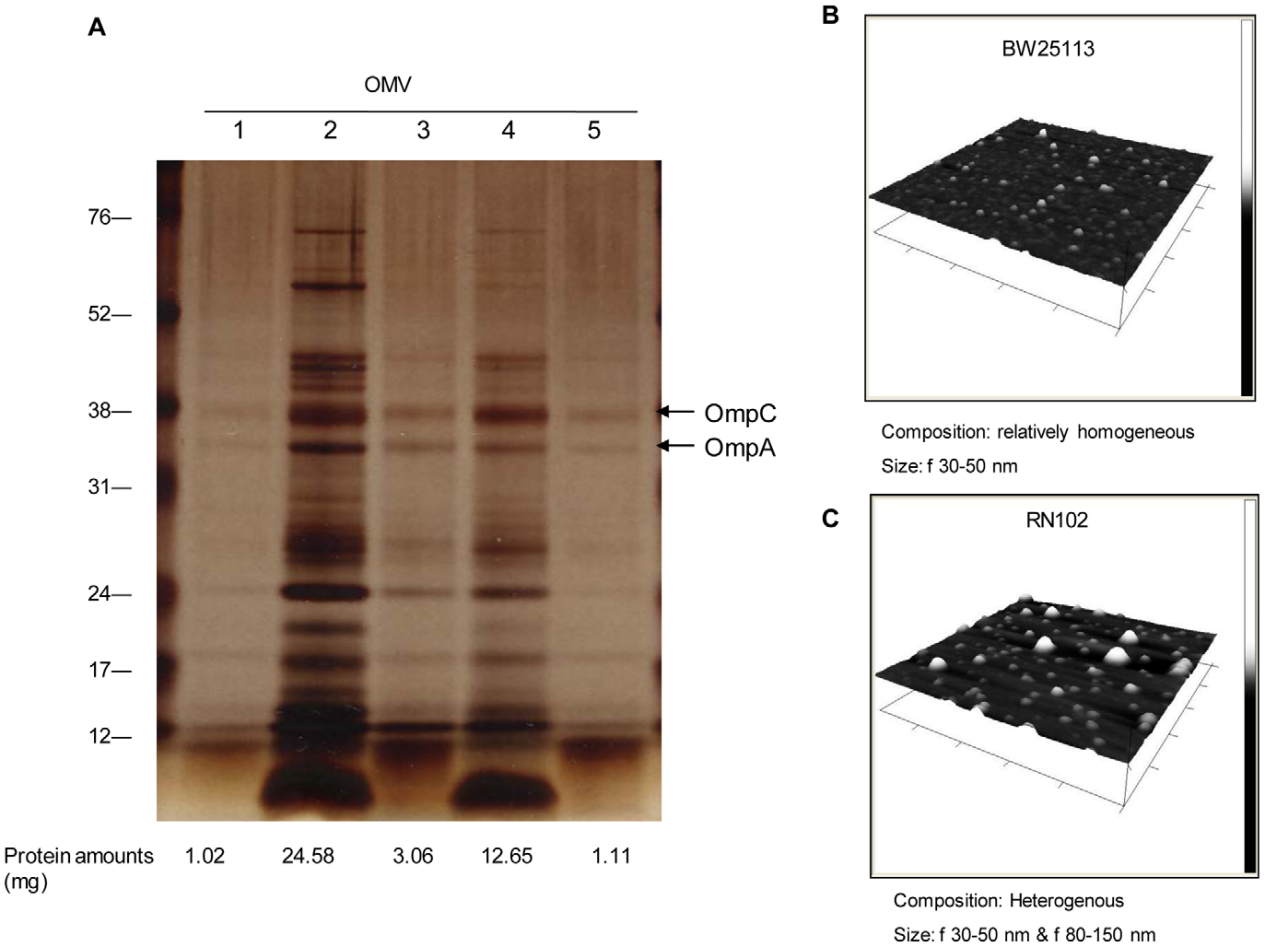
Analysis of OMV preparations. (A) The OMVs were isolated from the supernatant of a 80-ml bacterial liquid culture incubated 14 hours under shaking conditions. Finally, each OMV preparation was resuspended with 200 μl of 20 mM Tris-Cl (pH 8.0). Five μl of each OMV sample per well was run on 12% PAGE and subjected to silver staining. Total protein amounts (mg) of OMVs from a 80-ml bacterial culture of each strain were determined by Bradford assay and are presented below each lane. Lanes; 1, BW25113; 2, RN102; 3, BW25113/pNTR-SD; 4, RN102/pNTR-SD; 5, RN102/pNT3(*hldE*). (B and C) AFM images of OMVs prepared from BW25113 and RN102 on 1 μm^2^ surfaces were shown.

Interestingly, two major outer membrane proteins, OmpA and OmpC were also detected in the supernatant from the RN102, but not from the wild type (Fig. 7F). We also examined the presence of OMVs in the supernatants of these strains, because OMVs contain both outer membrane proteins and periplasmic proteins. OMVs isolated from the supernatants of the strains were analyzed on a SDS-PAGE gel and subjected to silver staining. Protein amounts of OMVs were quantified using Bradford assay (Fig. 8A), and morphology of OMVs was obserbed by atomic force microscopy (AFM) (Fig. 8B and C). Results of both silver staining and Bradford assay indicated that the amount of OMVs from the RN102 was much higher than that from the wild-type strain and in-*trans* complementation of the *hldE* mutation successfully restored the wild-type phenotype. (Fig. 8A). Bands of the two major outer membrane proteins of *E. coli*, OmpA and OmpC, were found in each lane at different density (shown by 2 arrows in Fig. 8A). The AFM-images of OMVs from BW25113 and the RN102 are shown in Figure 8B and C. While the morphology of OMVs obtained from the supernatant of the BW25113 strain was relatively homogeneous (30–50 nm in diameter), OMVs from of RN102 were more heterogeneous in size: two differently sized types of OMVs (30–50 nm and 80–150 nm in diameter) were observed. These data demonstrated that the *hldE* mutation also affected the morphology and amount of OMVs.

## Discussion

In the present study, we observed increased biofilm formation in some LPS core OS mutants, especially two deep rough LPS mutants, RN101 and RN102 (Fig. 2), which were the most aggregative strains among the series of LPS mutants used in this study (Fig. 3). These mutants were also the most hydrophobic among the strains, as determined by two different analyses: a bioassay using hexadecane (Fig. 4A) and a chemical analysis using XPS (Fig. 4B and S3). We suggest that the enhanced surface hydrophobicity of these mutant strains might lead to a strong tendency to autoaggregation and that the cell surface hydrophobicity *per se* determines the extent of adhesion to a hydrophobic substratum such as polystyrene. The observation is similar to recent reports concerning a mode of biofilm formation by a LPS mutant of *Porphyromonas gingivalis*, a *galE* mutant, which has truncated LPS and enhanced the autoaggregation and biofilm formation [14], [39] as well as hydrophobicity (Nakao et al., unpublished data). On the other hand, the *E. coli galE* mutant (RN107) examined in the present study was aggregative (Fig. 3) but it did not display an increased biofilm formation (Fig. 2) and did not show stronger hydrophobicity (Fig. 4). So, while it is to be expected that strong hydrophobicity is important for the bacterial attachment to abiotic surfaces, other effects than hydrophobicity may be involved in the autoaggregation in case of the RN107. In previous studies of the *galE* mutant of *P. gingivalis*, the pleiotropic effects occured in connection with changes in outer membrane components: increased OMV production, deglycosylation of LPS and an outer membrane protein, Omp85 homolog [39], [57], [58]. Therefore, some changes in components of outer membrane or bacterial appendage might be involved in the autoaggregative property of *galE* mutants in case of *E. coli* as well as the *P. gingivalis*. However, a more precise analysis is required to fully understand the mechanism of autoaggregation in RN107.

The cells of RN102 were found to be surrounded by EPS containing eDNA in biofilms (Fig. 6B and 6F, and Fig. S6) and DNase I treatment at the onset of the culture effectively prevented the biofilm formation (Fig. 6G and 6H). However, DNase I had no effect on matured biofilms. So, we suggest that eDNA might have a role not only as a predominant constituent of the mature biofilm structure, but also for the biofilm formation at early stages, perhaps for bacterial attachment and/or aggregation. On the other hand, the mechanism leading to an accumulation of eDNA in RN102 has not yet been determined. RN102 appeared to have a leaky outer membrane but still maintained the integrity of the inner membrane (Fig. 7F), suggesting that the eDNA accumulation occured by a mechanism other than lysis or total loss of membrane integrity. In *P. aeruginosa*, eDNA is released by lysis of bacterial cells and/or lysis of OMVs containing DNA within the lumen of the OMVs [37]; OMV is one of the major components of biofilms [36]. In case of RN102, the amount of *E. coli*-derived DNA in the supernatant is comparable to that of the wild type strain (Fig. 7G). We also detected a lot of dsDNA in the fresh LB media, which is expected to be derived from yeast extract. The amount of dsDNA (more than 500 ng/ml) might be saturated because an addition of DNA in the culture did not increase the biofilm formation (data not shown). A recent report showed that the presence of eDNA created highly hydrophobic bacterial cells, which contributed to initial attachment of bacteria and autoaggregation in the process of biofilm maturation [11]. So eDNA might confer hydrophobicity on the surface of RN102, resulting in enhanced biofilm formation. Analysis of the OMV preparation by SDS-PAGE and AFM (Fig. 8) demonstrated that strain RN102 produced more OMVs than the parental strain. Therefore, an increase in the amount of the OMVs in the case of RN102 may also contribute to the enhanced biofilm formation.

*E. coli* forms biofilms on abiotic surfaces in medical and industrial settings. In medical settings, the biofilms are frequently formed on indwelling medical devices such as urinary catheters or central venous catheters, resulting in medical device-associated infections. Biofilm formation on abiotic surfaces such as catheters or implants is different from that on biotic surfaces such as epithelium or endothelium. For example, protective host immunity against infections is less effective at abiotic surfaces than biotic surfaces because of the spatial distance from immunologically compent cells and molecules released from the host immune system. Therefore, bacteria generally survive and form biofilms easier on abiotic surfaces than the biotic surfaces. In addition, the medical devices such as catheters or implants are obliged to be often used for immunocompomised hosts. A deep rough mutant is generally considered to be less virulent and therefore inhibitors against core LPS biosynthesis have been considered good candidates for antibacterial development. However, in this study, we found that RN102 had enhanced biofilm formation with accumulation of eDNA. Therefore, it is possible that a deep rough phenotype can increase the risk of biofilm-associated infections. For example, in case of the polymicrobial infection, which frequently occurs in case of catheter-associated infections, a LPS core mutant might possibly enhance biofilm formation of other bacteria by means of its hydrophobic/aggregative phenotype and eDNA accumulation.

In conclusion, we demonstrated that mutations known to affect the composition of *E. coli* LPS core OS affected the biofilm formation which was associated with eDNA. We suggest the implication of the results should be considered in medical settings such as when there is risk for the catheter-associated infection, which is one of the serious and global problems in public health.

## Supporting Information

Figure S1 Recovery of O-antigen expression by introduction of the *wbbL* gene in-*trans*. The plasmid clone pMF19 and the vector control, pMF19Δ*wbbL* were introduced into two different *E. coli* K-12 strains, BW25113 and KP7600. Whole cells of each strain were analyzed on a 12% PAGE and subjected to silver staining. Lanes; 1, no plasmid; 2, pMF19; 3, pMF19Δ*wbbL* (vector control).
(DOC)

Figure S2 Biofilm formation by LPS O-antigen-expressing strains. The effect of O-antigen on biofilm formation was tested using two different background strains, BW25113 and KP7600. The mean ± SD of results from 3 independent experiments are shown. Statistical analysis was performed using Mann-Whitney's U test. *P<0.05 against biofilm formation level of strain BW25113.
(DOC)

Figure S3 XPS spectra of the LPS mutants. The aliphatic carbon components can be seen at 285 eV (arrows in Fig. S3A and B). When the ratio between this component and the others is increased this indicates increased hydrophobicity. Line coloring: (A) black, BW25113; light blue, RN101; red, RN102; pink, RN103; purple, RN104; light green, RN105; gray, RN106; broken brown, RN107. (B) black, BW25113; red, RN102; green, BW25113/pNTR-SD; blue, RN102/pNTR-SD; broken orange, RN102/pNT3(*hldE*).
(DOC)

Figure S4 Western blot analysis of FliC, OmpC, and Crp. Whole cells (Cell) and supernatants (Sup) were harvested from bacterial liquid cultures grown for 48 hours under static conditions. SDS-PAGE followed by Western blot analysis using anti-FliC, OmpC, and Crp antisera was performed. Band density analysis from Western blots signals (FliC and OmpC) in the whole cells sample are shown as ratios of FliC/Crp and OmpC/Crp in the panels on the right side. Lanes; 1, BW25113; 2, RN101; 3, RN102; 4, RN103; 5, RN104; 6, RN105; 7, RN106; 8, RN107; 9, RN110.
(DOC)

Figure S5 Ag43 overexpression in RN102 and analysis of the *hldE agn43* double mutant, RN109. (A and B) Whole cells were harvested from bacterial liquid cultures grown for 48 hours under static conditions. SDS-PAGE followed by Western blot analysis using anti-Ag43 antiserum and OmpA antiserum (as a loading control) was performed. Lanes; (A) 1, BW25113; 2, RN102; 3, BW25113/pNTR-SD; 4, RN102/pNTR-SD; 5, RN102/pNT3(hldE); 6, MG1655 *agn43* (negative control); 7, MG1655 *oxyR* (positive control); (B) 1, RN108; 2, RN109; 3, blank; 4, MG1655 *oxyR* (positive control). (C and D) Autoaggregation and biofilm formation of strains BW25113, RN102, RN108, and RN109. (C) The value of autoaggregation is shown as the mean ± SD of results from three independent experiments. (D) Biofilm formation by RN109 when compared to the parental strains. The results are shown as the mean ± SD of a quadruplicate assay.
(DOC)

Figure S6 eDNA in biofilms of BW25113 and RN102. (A) Shown are two-dimension CLSM images of biofilms stained with SYTO 9 (green) and BOBO-3 (red), which allow visualization of cells and eDNA, respectively. Single-color (green and red) and merged images are shown. Scale bars represent 40 μm for all panels. Images represent single optical sections acquired in comparable focal planes of the three-dimensional structure of the biofilms. (B) eDNAs associated with biofilms of BW25113 and RN102 strains were run on 1% agarose gel. An arrow indicates the chromosomal DNA at high molecular weight.
(DOC)

Table S1 Oligonucleotides used in this study.
(XLS)
