# Supplementary material for: Enhanced Biofilm Formation by Escherichia coli LPS Mutants Defective in Hep Biosynthesis
Source: PLoS One. 2012 Dec 28;7(12):e51241. doi: 10.1371/journal.pone.0051241 (PMC3532297; doi:10.1371/journal.pone.0051241)
Supplement: Figure S1 — Recovery of O-antigen expression by introduction of the wbbL gene in-trans. The plasmid clone pMF19 and the vector control, pMF19ΔwbbL were introduced into two different E. coli K-12 strains, BW25113 and KP7600. Whole cells of each strain were analyzed on a 12% PAGE and subjected to silver staining. Lanes; 1, no plasmid; 2, pMF19; 3, pMF19ΔwbbL (vector control). (DOC) [file pone.0051241.s001.doc]

**
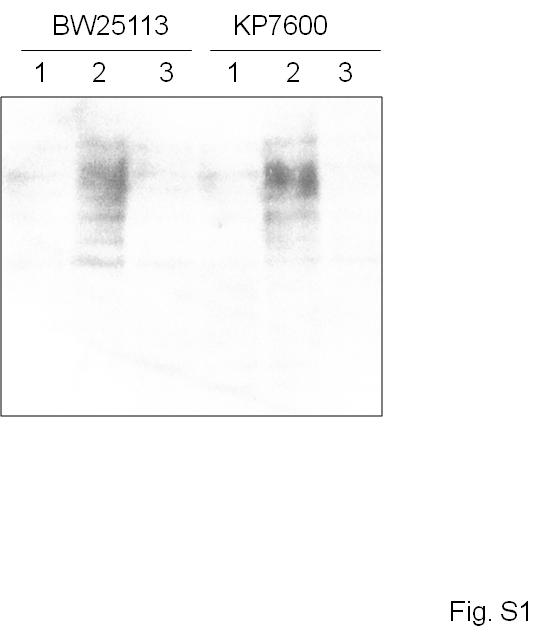
**

**Figure S1. Recovery of O-antigen expression by introduction of the *wbbL* gene in-*trans*.** The plasmid clone pMF19 and the vector control, pMF19Δ*wbbL* were introduced into two different *E. coli* K-12 strains, BW25113 and KP7600. Whole cells of each strain were analyzed on a 12 % PAGE and subjected to silver staining. Lanes; 1, no plasmid; 2, pMF19; 3, pMF19Δ*wbbL* (vector control).
