# Supplementary material for: Enhanced Biofilm Formation by Escherichia coli LPS Mutants Defective in Hep Biosynthesis
Source: PLoS One. 2012 Dec 28;7(12):e51241. doi: 10.1371/journal.pone.0051241 (PMC3532297; doi:10.1371/journal.pone.0051241)
Supplement: Figure S2 — Biofilm formation by LPS O-antigen-expressing strains. The effect of O-antigen on biofilm formation was tested using two different background strains, BW25113 and KP7600. The mean ± SD of results from 3 independent experiments are shown. Statistical analysis was performed using Mann-Whitney's U test. *P<0.05 against biofilm formation level of strain BW25113. (DOC) [file pone.0051241.s002.doc]

**
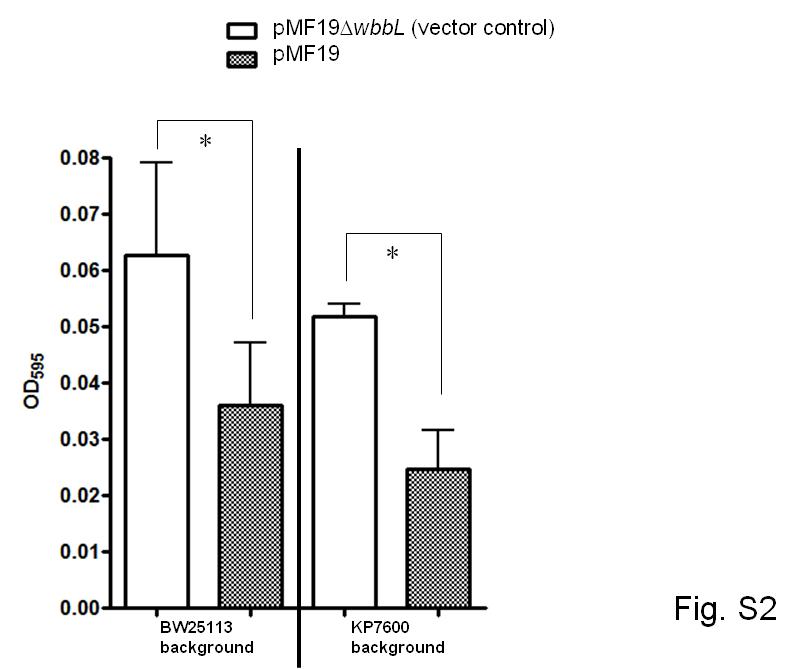
**

**Figure S2. Biofilm formation by LPS O-antigen-expressing strains.** The effect of O-antigen on biofilm formation was tested using two different background strains, BW25113 and KP7600. The mean ± SD of results from 3 independent experiments are shown. Statistical analysis was performed using Mann-Whitney’s U test. **P*<0.05 against biofilm formation level of strain BW25113.
