# Supplementary material for: Enhanced Biofilm Formation by Escherichia coli LPS Mutants Defective in Hep Biosynthesis
Source: PLoS One. 2012 Dec 28;7(12):e51241. doi: 10.1371/journal.pone.0051241 (PMC3532297; doi:10.1371/journal.pone.0051241)
Supplement: Figure S3 — XPS spectra of the LPS mutants. The aliphatic carbon components can be seen at 285 eV (arrows in Fig. S3A and B). When the ratio between this component and the others is increased this indicates increased hydrophobicity. Line coloring: (A) black, BW25113; light blue, RN101; red, RN102; pink, RN103; purple, RN104; light green, RN105; gray, RN106; broken brown, RN107. (B) black, BW25113; red, RN102; green, BW25113/pNTR-SD; blue, RN102/pNTR-SD; broken orange, RN102/pNT3(hldE). (DOC) [file pone.0051241.s003.doc]

**
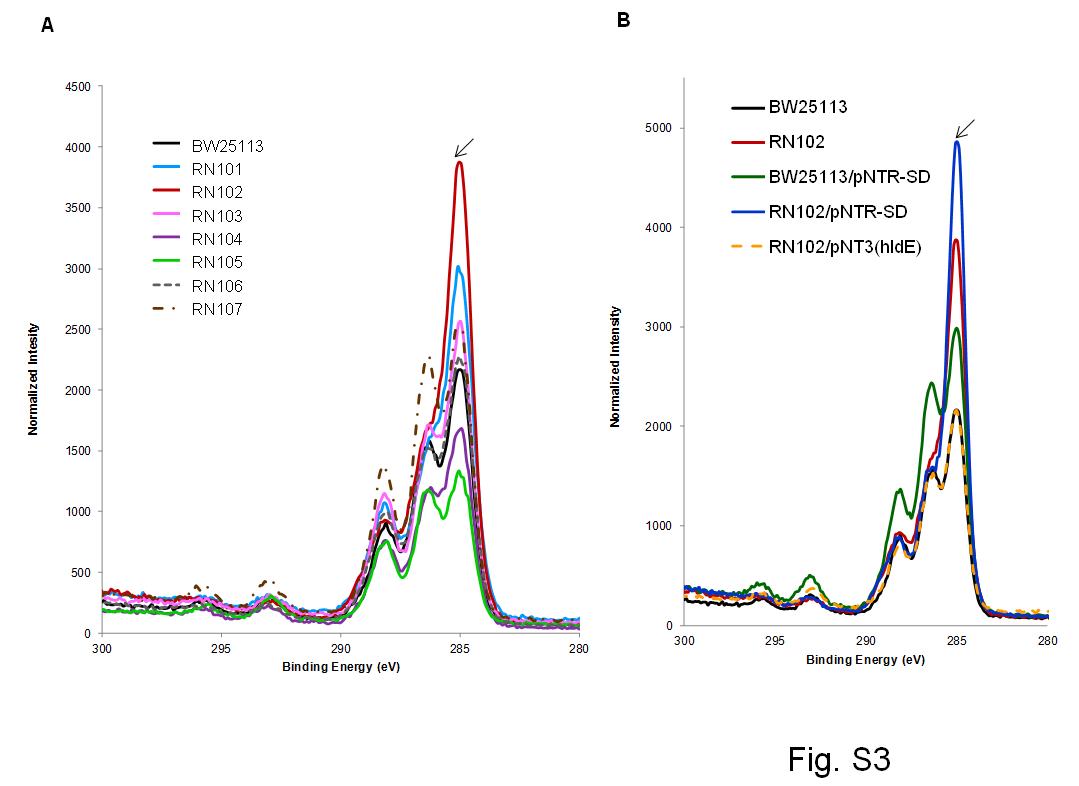
**

**Figure S3. XPS spectra of the LPS mutants.** The aliphatic carbon components can be seen at 285 eV (arrows in Fig. S3A and B). When the ratio between this component and the others is increased this indicates increased hydrophobicity. Line coloring: (A) black, BW25113; light blue, RN101; red, RN102; pink, RN103; purple, RN104; light green, RN105; gray, RN106; broken brown, RN107. (B) black, BW25113; red, RN102; green, BW25113/pNTR-SD; blue, RN102/pNTR-SD; broken orange, RN102/pNT3(*hldE*).
