# Supplementary material for: Enhanced Biofilm Formation by Escherichia coli LPS Mutants Defective in Hep Biosynthesis
Source: PLoS One. 2012 Dec 28;7(12):e51241. doi: 10.1371/journal.pone.0051241 (PMC3532297; doi:10.1371/journal.pone.0051241)
Supplement: Figure S4 — Western blot analysis of FliC, OmpC, and Crp. Whole cells (Cell) and supernatants (Sup) were harvested from bacterial liquid cultures grown for 48 hours under static conditions. SDS-PAGE followed by Western blot analysis using anti-FliC, OmpC, and Crp antisera was performed. Band density analysis from Western blots signals (FliC and OmpC) in the whole cells sample are shown as ratios of FliC/Crp and OmpC/Crp in the panels on the right side. Lanes; 1, BW25113; 2, RN101; 3, RN102; 4, RN103; 5, RN104; 6, RN105; 7, RN106; 8, RN107; 9, RN110. (DOC) [file pone.0051241.s004.doc]

**
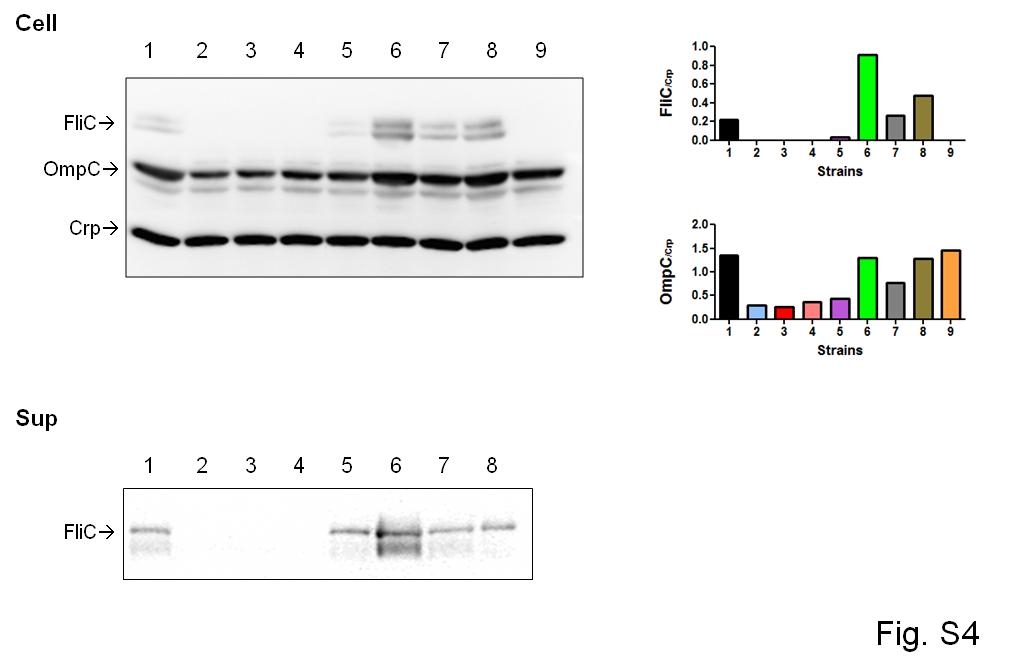
**

**Figure S~~4~~. Western blot analysis of FliC, OmpC, and Crp.** Whole cells (Cell) and supernatants (Sup) were harvested from bacterial liquid cultures grown for 48 hours under static conditions. SDS-PAGE followed by Western blot analysis using anti-FliC, OmpC, and Crp antisera was performed. Band density analysis from Western blots signals (FliC and OmpC) in the whole cells sample are shown as ratios of FliC/Crp and OmpC/Crp in the panels on the right side. Lanes; 1, BW25113; 2, RN101; 3, RN102; 4, RN103; 5, RN104; 6, RN105; 7, RN106; 8, RN107; 9, RN110.
