# Supplementary material for: Enhanced Biofilm Formation by Escherichia coli LPS Mutants Defective in Hep Biosynthesis
Source: PLoS One. 2012 Dec 28;7(12):e51241. doi: 10.1371/journal.pone.0051241 (PMC3532297; doi:10.1371/journal.pone.0051241)
Supplement: Figure S5 — Ag43 overexpression in RN102 and analysis of the hldE agn43 double mutant, RN109. (A and B) Whole cells were harvested from bacterial liquid cultures grown for 48 hours under static conditions. SDS-PAGE followed by Western blot analysis using anti-Ag43 antiserum and OmpA antiserum (as a loading control) was performed. Lanes; (A) 1, BW25113; 2, RN102; 3, BW25113/pNTR-SD; 4, RN102/pNTR-SD; 5, RN102/pNT3(hldE); 6, MG1655 agn43 (negative control); 7, MG1655 oxyR (positive control); (B) 1, RN108; 2, RN109; 3, blank; 4, MG1655 oxyR (positive control). (C and D) Autoaggregation and biofilm formation of strains BW25113, RN102, RN108, and RN109. (C) The value of autoaggregation is shown as the mean ± SD of results from three independent experiments. (D) Biofilm formation by RN109 when compared to the parental strains. The results are shown as the mean ± SD of a quadruplicate assay. (DOC) [file pone.0051241.s005.doc]

**
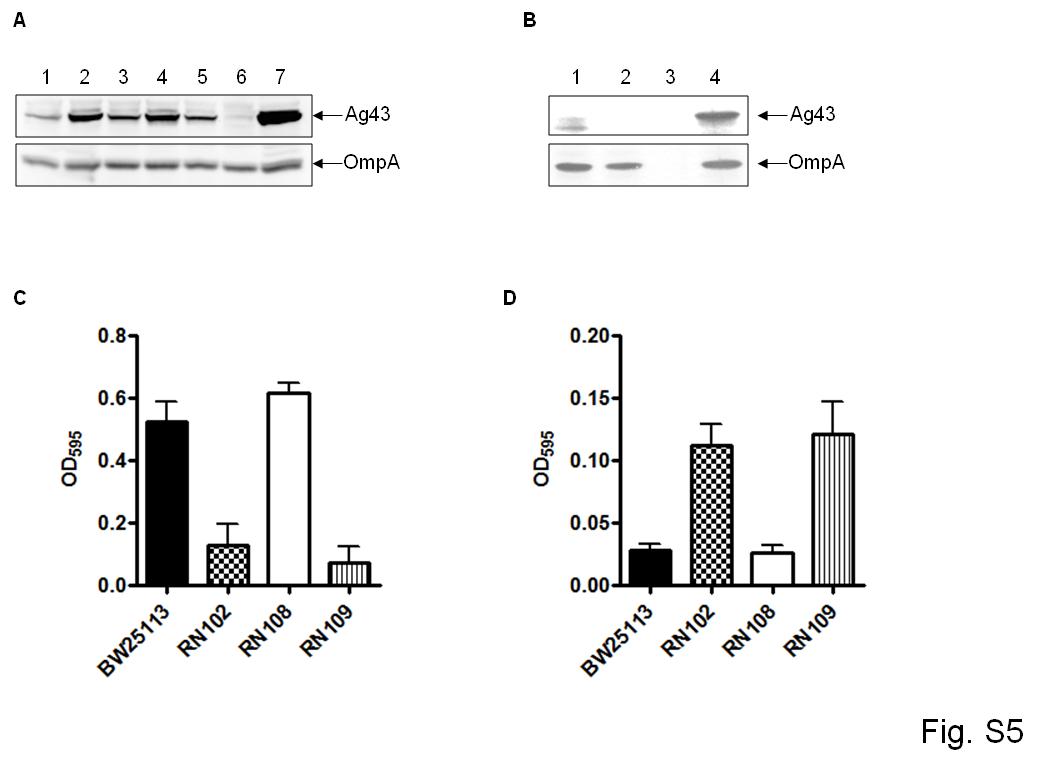
**

**Figure S5. Ag43 overexpression in RN102 and analysis of the *hldE agn43* double mutant, RN109.** (A and B) Whole cells were harvested from bacterial liquid cultures grown for 48 hours under static conditions. SDS-PAGE followed by Western blot analysis using anti-Ag43 antiserum and OmpA antiserum (as a loading control) was performed. Lanes; (A) 1, BW25113; 2, RN102; 3, BW25113/pNTR-SD; 4, RN102/pNTR-SD; 5, RN102/pNT3(hldE); 6, MG1655 *agn43* (negative control); 7, MG1655 *oxyR* (positive control); (B) 1, RN108; 2, RN109; 3, blank; 4, MG1655 *oxyR* (positive control). (C and D) Autoaggregation and biofilm formation of strains BW25113, RN102, RN108, and RN109. (C) The value of autoaggregation is shown as the mean ± SD of results from three independent experiments. (D) Biofilm formation by RN109 when compared to the parental strains. The results are shown as the mean ± SD of a quadruplicate assay.
