# Supplementary material for: Enhanced Biofilm Formation by Escherichia coli LPS Mutants Defective in Hep Biosynthesis
Source: PLoS One. 2012 Dec 28;7(12):e51241. doi: 10.1371/journal.pone.0051241 (PMC3532297; doi:10.1371/journal.pone.0051241)
Supplement: Figure S6 — eDNA in biofilms of BW25113 and RN102. (A) Shown are two-dimension CLSM images of biofilms stained with SYTO 9 (green) and BOBO-3 (red), which allow visualization of cells and eDNA, respectively. Single-color (green and red) and merged images are shown. Scale bars represent 40 μm for all panels. Images represent single optical sections acquired in comparable focal planes of the three-dimensional structure of the biofilms. (B) eDNAs associated with biofilms of BW25113 and RN102 strains were run on 1% agarose gel. An arrow indicates the chromosomal DNA at high molecular weight. (DOC) [file pone.0051241.s006.doc]

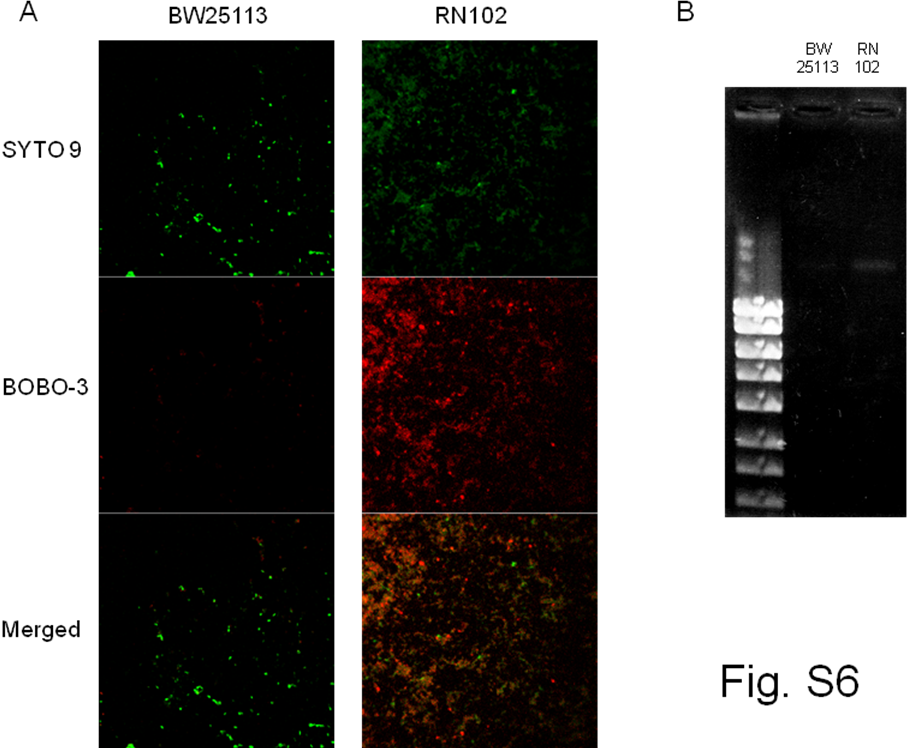


(kb)

10

8

6

5

4

3

2.5

2

**Figure S6. eDNA in biofilms of BW25113 and RN102.**  (A) Shown are two-dimension CLSM images of biofilms stained with SYTO 9 (green) and BOBO-3 (red), which allow visualization of cells and eDNA, respectively. Single-color (green and red) and merged images are shown. Scale bars represent 40 μm for all panels. Images represent single optical sections acquired in comparable focal planes of the three-dimensional structure of the biofilms. (B) eDNAs associated with biofilms of BW25113 and RN102 strains were run on 1% agarose gel. An arrow indicates the chromosomal DNA at high molecular weight.
